# Investigation of Fungal Community Structure in the Gut of the Stag Beetle *Dorcus hopei* (Coleoptera; Lucanidae): Comparisons Among Developmental Stages

**DOI:** 10.1007/s00248-024-02379-y

**Published:** 2024-05-14

**Authors:** Xiaoyan Bin, Pan Wang, Yagang Shen, Xingjia Xiang, Muhammad Jafir, Xia Wan

**Affiliations:** 1 School of Resources and Environmental Engineering, Anhui University, Hefei, 230601 China; 2 Anhui Province Key Laboratory of Wetland Ecosystem Protection and Restoration, Hefei, 230601 China

**Keywords:** Fungivory, Saproxylic insects, Intestinal fungal, Life stages

## Abstract

Stag beetles, recognized as common saproxylic insects, are valued for their vibrant coloration and distinctive morphology. These beetles play a crucial ecological role in decomposition and nutrient cycling, serving as a vital functional component in ecosystem functioning. Although previous studies have confirmed that stag beetles are predominantly fungivores, the fluctuations in their intestinal fungal communities at different developmental stages remain poorly understood. In the current study, high-throughput sequencing was employed to investigate the dynamic changes within intestinal fungal communities at various developmental stages in the stag beetle *Dorcus hopei*. Results showed that microbial diversity was higher during the larval stage than during the pupal and adult stages. Furthermore, significant differences were identified in the composition of the intestinal fungal communities across the larval, pupal, and adult stages, suggesting that developmental transitions may be crucial factors contributing to variations in fungal community composition and diversity. Dominant genera included *Candida*, *Scheffersomyces*, *Phaeoacremonium*, and *Trichosporon*. Functional predictions indicated a greater diversity and relative abundance of endosymbiotic fungi in the larval gut, suggesting a potential dependency of larvae on beneficial gut fungi for nutrient acquisition. Additionally, the application of abundance-based β-null deviation and niche width analyses revealed that the adult gut exerted a stronger selection pressure on its fungal community, favoring certain taxa. This selection process culminates in a more robust co-occurrence network of fungal communities within the adult gut, thereby enhancing their adaptability to environmental fluctuations. This study advances our understanding of the intestinal fungal community structure in stag beetles, providing a crucial theoretical foundation for the development of saproxylic beetle resources, biomass energy utilization, plastic degradation strategies, and beetle conservation efforts.

**Supplementary Information:**

The online version contains supplementary material available at 10.1007/s00248-024-02379-y.

## Introduction

Beetles, belonging to the order Coleoptera, represent one of the most abundant and diverse groups of insects, accounting for approximately 25% of all known animal species [1, 2]. Within Coleoptera, members of the family Lucanidae are distinguished not only for their striking morphological features, such as enlarged mandibles that resemble deer antlers, but also for their pivotal role in forest ecosystems as saproxylic decomposers of woody debris [3–5]. Lucanid beetles also possess a highly specialized and complex gut system, which is home to a vast array of microbes [6, 7] and plays a pivotal role in facilitating insect adaptation to various environments and food resources, including the acquisition of essential amino acids, vitamins, and sterols [8–11]. Stag beetles, as prominent representatives of the Lucanidae family, exemplify a profound symbiosis between insects and their gut microbiota. During their different developmental stages, stag beetles utilize intestinal microbes (both bacteria and fungi) to maintain health and digest food, especially otherwise indigestible macromolecular substances [12, 13]. This symbiotic relationship highlights the potential of these microbes in nutrient recycling, as well as industrial applications such as plastic degradation and enzyme production [14–16]. Understanding the dynamics of gut microbes is essential for devising informed conservation strategies, which are crucial for maintaining beetle diversity and ecosystem resilience in response to climate change.

With the microbial communities residing in insect guts, fungi have garnered increasing attention due to their significant impact on insect physiology and ecology [8, 9]. Gut fungi serve as integral components of the digestive systems of various insect species, facilitating the decomposition of refractory macromolecular organic matter in food and providing essential nutrients necessary for host survival [17]. Additionally, gut fungi enhance symbiotic relationships between insects and their microbial partners, impacting host development, fitness, and ecological interactions. They also produce various enzymes, such as cellulases, peroxidases, laccases, xylanases, and xyloglucanases, which help in the degradation of cellulose, lignin, and hemicellulose, thus playing a pivotal mutualistic role in stag beetle guts [18–21]. By facilitating the decomposition and assimilation of complex plant-derived carbohydrates and nitrogenous substances, which insects typically find difficult to decompose, gut fungi can help overcome dietary limitations associated with nutrient-poor food sources, thus supporting beetle development and reproduction [22]. Furthermore, these gut-residing fungi contribute to ecosystem nutrient cycling by transforming low-nutrition food sources into bioavailable nutrients [17].

While previous research has successfully reared the fungivorous stag beetle *Dorcus rectus* (Motschulsky) in controlled laboratory environments [23], whether *Dorcus hopei* can complete its life cycle on a fungal diet in laboratory settings has not been reported. Examination of the interactions between fungivorous diets and different life stages of *D. hopei* could help elucidate the distribution and abundance of this insect and provide insights into the ecological and physiological importance of fungal microbes in *D. hopei* biology. This study selected the giant stag beetle at different developmental stages (1st, 2nd, and 3rd instar larval, pupa, callow adult, mature adult) under fungivorous conditions as the research subject. High-throughput Illumina MiSeq sequencing was employed to investigate the diversity and composition of the intestinal fungal community, exploring its potential role in the growth and development of *D. hopei*. Specifically, we aimed to (1) reveal the dynamic changes in fungal communities throughout the developmental stages of *D*. *hopei* under artificial diets, (2) characterize fungal community diversity in adults and larvae fed two distinct diets, and (3) explore the distribution and assembly of core fungi within the *D*. *hopei* gut.

## Materials and Methods

### Sample Collection and Rearing Conditions

The *D. hopei* stag beetles were sourced from the Mu-Ye Insect Company (Lishui, Zhejiang, China) and subsequently reared in the laboratory. In total, 72 specimens were artificially reared under optimum conditions (temperature: 22–25 °C). The larvae were fed an artificial diet containing fermented wood shavings mixed with fungal mycelia from *Pleurotus geesteranus* in pudding boxes (fermented *Quercus acutissima* wood), which was continuously replaced and rehydrated. Adults were fed beetle jelly. At different developmental stages, including larvae (1st (L1), 2nd (L2), and 3rd (L3) instars, with 13 individuals for each instar), pupae (Pu (12 individuals)), callow adults (CAd (eight individuals)), and mature adults (MAd (13 individuals)), specimens were collected for analysis.

#### Sample Dissection and Microbial DNA Extraction

Before dissection, all samples were disinfected for 3 min with 70% ethanol, followed by washing with distilled water to clean the surface. The samples were then treated with tenfold diluted phosphate-buffered saline (PBS) (total 500 mL, NaCl 1.37 M, KCl 26.8 mM, Na_2_HPO_4_ 81.0 mM, KH_2_PO_4_ 17.6 mM, pH 7.2–7.4) on a horizontal clean bench [24]. The midgut and hindgut were removed using sterile fine-tip forceps and placed into 2 mL of Lysing Matrix E under sterile conditions to avoid contamination. Microbial DNA from the gut of *D. hopei* was extracted using a Fast DNA® SPIN for Soil Kit (MP Biomedicals, Solon, OH, USA), following the provided instructions, then stored at − 20 °C before use.

### Polymerase Chain Reaction (PCR) Amplification

Purified DNA from each sample was utilized as a template for amplification, with the primer pairs ITS1F (5′-CTTGGTCATTTAGAGGAAGTAA-3′) [25] and ITS2R (5′-GCTGCGTTCTTCATCGATGC-3′) [26] used for PCR amplification of fungal internal transcribed spacer (ITS) fragments. The ITS region is recommended as the universal DNA bar-code marker for fungal identification [27]. The PCR procedures were carried out in 20-μL reaction mixtures containing 2 μL of 10 × buffer, 2 μL of 2.5 mM dNTPs, 0.8 μL of each primer, 0.2 μL of TaKaRa rTaq DNA polymerase, 0.2 μL of bovine serum albumin (BSA), 10 ng of template DNA, and deionized water (to 20 μL). The thermal cycling for PCR included an initial step at 95 °C for 3 min, followed by 27 cycles at 95 °C for 30 s, 55 °C for 30 s, and 72 °C for 45 s, with a final extension at 72 °C for 10 min. Negative controls without DNA template were included to check for potential contamination. Amplification products were detected by 2% agarose gel electrophoresis, and subsequent sequencing of the amplified PCR products was conducted by Majorbio (Shanghai, China).

### Processing of Sequencing Data

The raw data were processed for preliminary analysis using QIIME v.1.9 [28]. Sequences with the same barcode primer were first grouped into one sample. The sequences obtained from high-throughput sequencing were spliced, the number of overlapping bases was set to be no less than 20, and the error rate of base pairing was 0. After splicing, low-quality sequences (average quality score < 30, length < 250 bp) were removed, ensuring a q-value of sample sequences above 25. The high-quality sequences were saved to seqs.fna, and chimeras were removed with USEARCH (v1. 8.0). Subsequently, the sequences were subjected to cluster analysis, and high-quality sequences were clustered into operational taxonomic units (OTUs) based on 97% similarity using the de novo approach. Representative ITS sequences from these OTUs were then classified taxonomically using the UNITE v.8.0 (http://unite.ut.ee) database [29]. To equally rarefy samples, randomly selected subsets of 3000 sequences (lowest sequence read depth; repetition 20 times) per sample were used to compare fungal community composition and diversity for all samples.

### Statistical Analysis

Fungal alpha diversity and relative abundance of dominant genera among life stages were based on one-way analysis of variance (ANOVA) using the SPSS v20.0 (Chicago, IL, USA) [30]. Differences in fungal community composition between different stages and sexes were analyzed by non-metric multidimensional scaling (NMDS) and analysis of similarity (ANOSIM; permutations = 999) using the vegan package in R (v4.3.0) [31–33]. The contribution of fungal OTUs to differences among stages was analyzed using SIMPER in the vegan package in R (v4.3.0) [32]. Biomarkers of intestinal fungi in different developmental stages were identified by linear discriminant analysis (LDA) effect size (LEfSe) [33]. Indicator analysis was performed using the labdsv package in R (v4.3.0). The FUNGuild method was used to predict fungal community functions, with only those showing high confidence (i.e., highly probable and possible) selected for further analysis, visualized using the Sigmaplot (v12.5) [34]. The abundance-based beta-null model was used to differentiate the relative significance of deterministic and stochastic processes based on null deviation (NDV) [35]. Co-occurrence network analyses were conducted using the dplyr, igraph, and Hmisc packages in R (v4.3.0) and visualized in Gephi (v0.9.2) [36].

## Results

### Intestinal Fungal Alpha Diversity

In this study, a total of 3,717,848 high-quality sequences were obtained after processing 72 fungal samples, ranging from 3105 to 110,938 sequences per sample. Venn diagram analysis identified 2357 fungal OTUs, including 631, 713, and 405 OTUs in larvae, pupae, and adults, respectively. Additionally, unique OTUs were identified at each developmental stage, with adults containing 210 unique OTUs, representing 15.3% of total OTUs identified in adults, larvae containing a higher proportion of unique OTUs, accounting for 28.2%, and pupae exhibiting the highest proportion of unique OTUs at 36.3%. Venn diagram analysis was used to compare the similarities and differences between the communities at different developmental stages (Fig. S1).

**Fig. 1:**
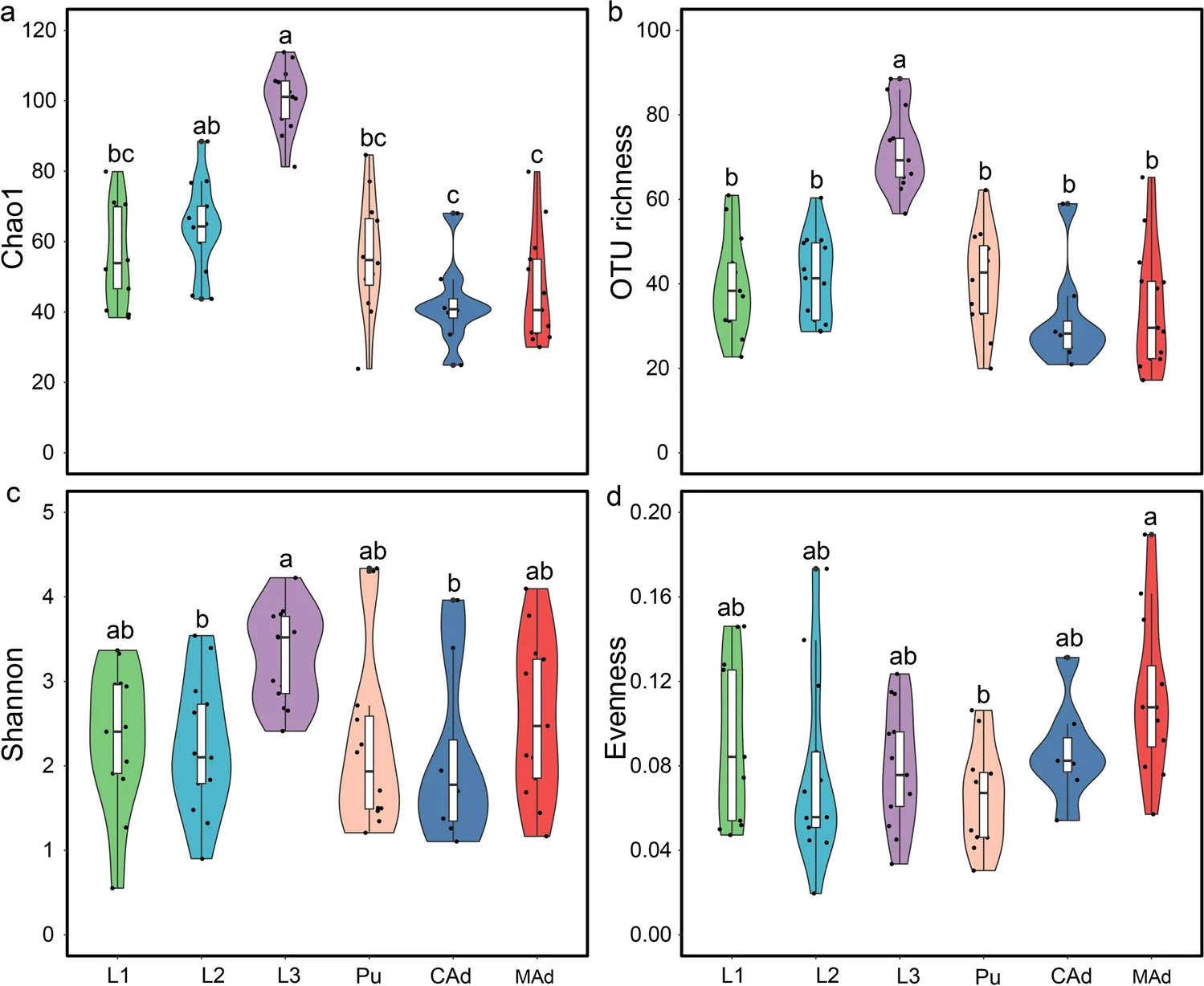
Intestinal fungal alpha diversity at different developmental stages, including Chao1 (**a**), OTU richness (**b**), Shannon (**c**), Evenness (**d**). Different letters above bars represent significant differences based on Tukey’s HSD comparisons (*P* < 0.05). 1st instar (L1), 2nd instar (L2), 3rd instar (L3), pupa (Pu), callow adult (CAd), and mature adult (MAd)

The *D. hopei* stag beetle undergoes complete metamorphosis, leading to significant differences in diet and function between larvae and adults. In this study, we analyzed four alpha diversity indices (OTU richness, Shannon, Evenness, and Chao1) to evaluate variations in gut fungal diversity across six developmental stages and among insects subjected to two distinct diets. Results showed that fungal alpha diversity initially increased, then decreased, and subsequently increased again, with the highest diversity observed in 3rd instar larvae and the lowest in callow adults (Fig. 1).

### Intestinal Fungal Community Structure

The observed variations in beta diversity were attributable to differences in the community structure of gut fungi. The dominant genera, including *Scheffersomyces* (38.23%), *Phaeoacremonium* (30.43%), *Trichosporon* (8.55%), and *Candida* (4.19%), showed marked differences in their relative abundances across larvae, pupae, and adults (one-way ANOVA: *P* < 0.05). Similarly, significant differences were noted among the 1st instar, 2nd instar, 3rd instar, callow adult, and mature adult stages (one-way ANOVA: *P* < 0.05).

**Fig. 2:**
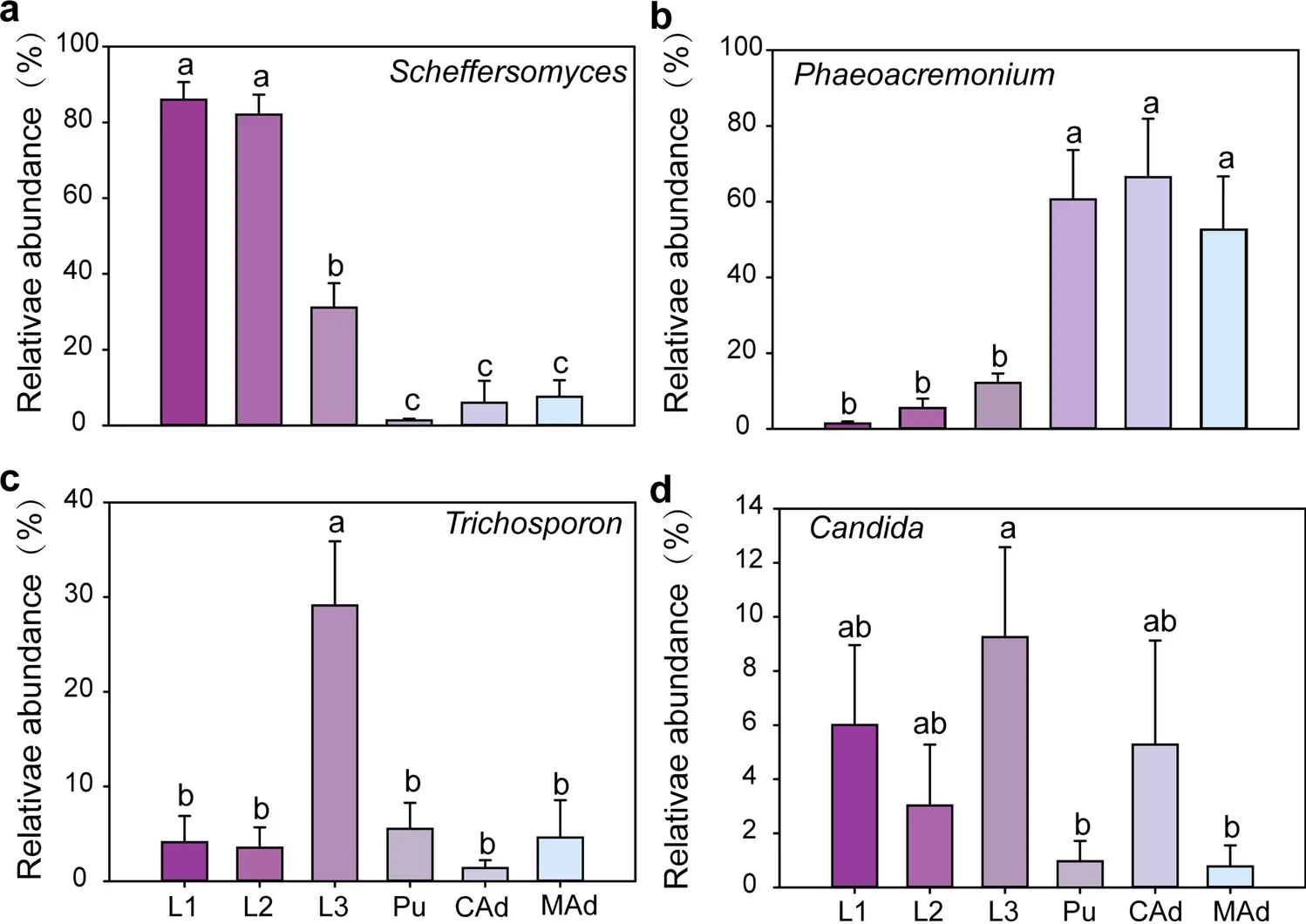
Relative abundance of fungal taxa at the genus level in the gut of *D*. *hopei* at different life stages, respectively are *Scheffersomyces* (**a**), *Phaeoacremonium* (**b**), *Trichosporon* (**c**), *Candida* (**d**). Bars represent mean, error bars denote standard deviation, and letters above bars represent significant differences from one-way ANOVA (*P* < 0.05). 1st instar (L1), 2nd instar (L2), 3rd instar (L3), pupa (Pu), callow adult (CAd), and mature adult (MAd)

The relative abundance of *Scheffersomyces* was significantly higher in 1st and 2nd instar larvae compared to the other developmental stages. *Phaeoacremonium* displayed significantly higher relative abundance during the pupal and adult stages than during the larval stage. The peak relative abundance of *Trichosporon* was highest in 3rd instar larvae compared to other stages. Furthermore, *Candida* was also significantly higher in the 3rd instar stage compared to the other developmental stages (Fig. 2).

**Fig. 3:**
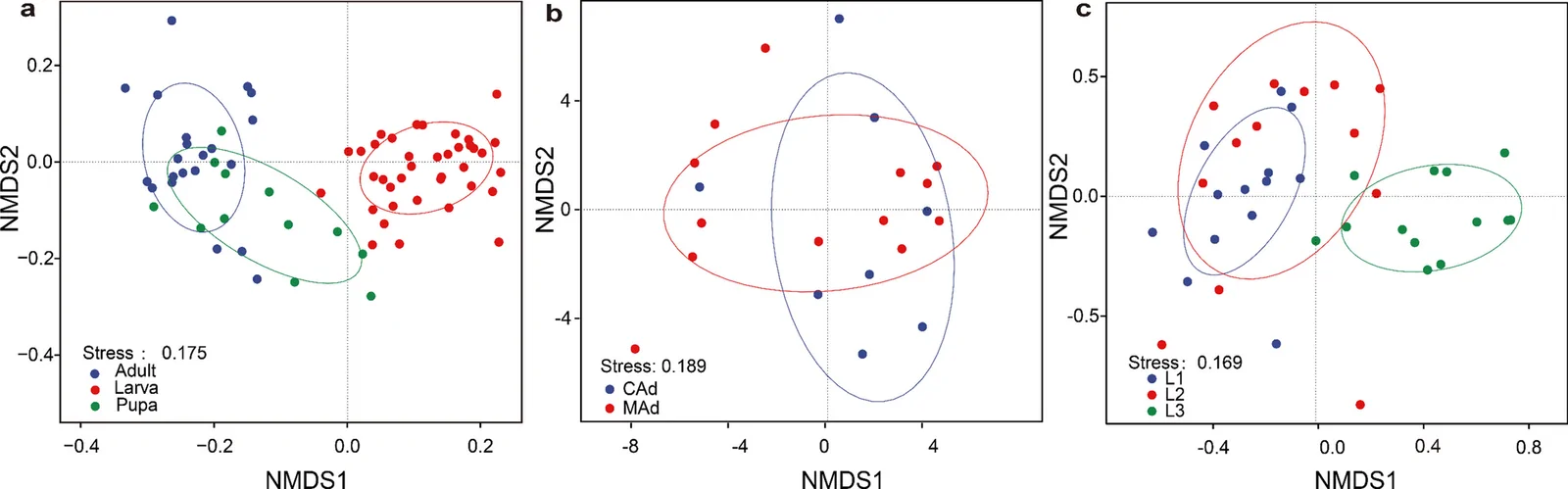
NMDS plots showing fungal community composition in larvae, pupae, and adults (**a**), between different adult stages (**b**), and between different larval stages (**c**). 1st instar (L1), 2nd instar (L2), 3rd instar (L3), pupa (Pu), callow adult (CAd), and mature adult (MAd)

The NMDS and ANOSIM analyses revealed distinct fungal community structures across the various developmental stages of *D. hopei*, with significant differences observed in larvae, pupae, and adults (ANOSIM: *P* = 0.001; Table 1; Fig. 3). Notably, the fungal communities in the 1st instar larvae differed markedly from those in the 2nd (*P* = 0.039) and 3rd instar larvae (*P* = 0.001) (Fig. 3c). In contrast, based on Tukey’s HSD test for multiple comparisons, the community composition between callow and mature adults showed a similar fungal structure (Fig. 3b; Table 1).

**Table 1:** Differences in fungal community composition at different developmental stages based on ANOSIM. 1st instar (L1), 2nd instar (L2), 3rd instar (L3), pupa (Pu), callow adult (CAd), and mature adult (MAd)

Stage	ANOSIM	
	*r*	*P*
L1 *vs* L2	− 0.034	0.716
L1 *vs* L3	0.257	0.005
L1 *vs* Pu	0.565	0.001
L1 *vs* CAd	0.679	0.005
L1 *vs* MAd	0.371	0.001
L2 *vs* L3	0.186	0.007
L2 *vs* Pu	0.403	0.001
L2 *vs* CAd	0.554	0.001
L2 *vs* MAd	0.237	0.005
L3 *vs* Pu	0.278	0.001
L3 *vs* CAd	0.460	0.001
L3 *vs* MAd	0.186	0.006
Pu *vs* CAd	0.084	0.123
Pu *vs* MAd	0.055	0.147
CAd *vs* MAd	− 0.096	0.938
Larva *vs* Pupa	0.718	0.001
Larva *vs* Adult	0.523	0.001
Pupa *vs* Adult	0.013	0.338

The neutral abundance-based β-null model was applied to differentiate the relative importance of deterministic and stochastic processes in the assembly of intestinal fungal communities at different developmental stages (Fig. S2a). Results showed an increasing trend in NDV, suggesting a greater influence of deterministic processes across developmental stages. Additionally, habitat niche breadth values of intestinal fungal communities demonstrated a marked decrease with advancing developmental stages (Fig. S2b).

**Fig. 4:**
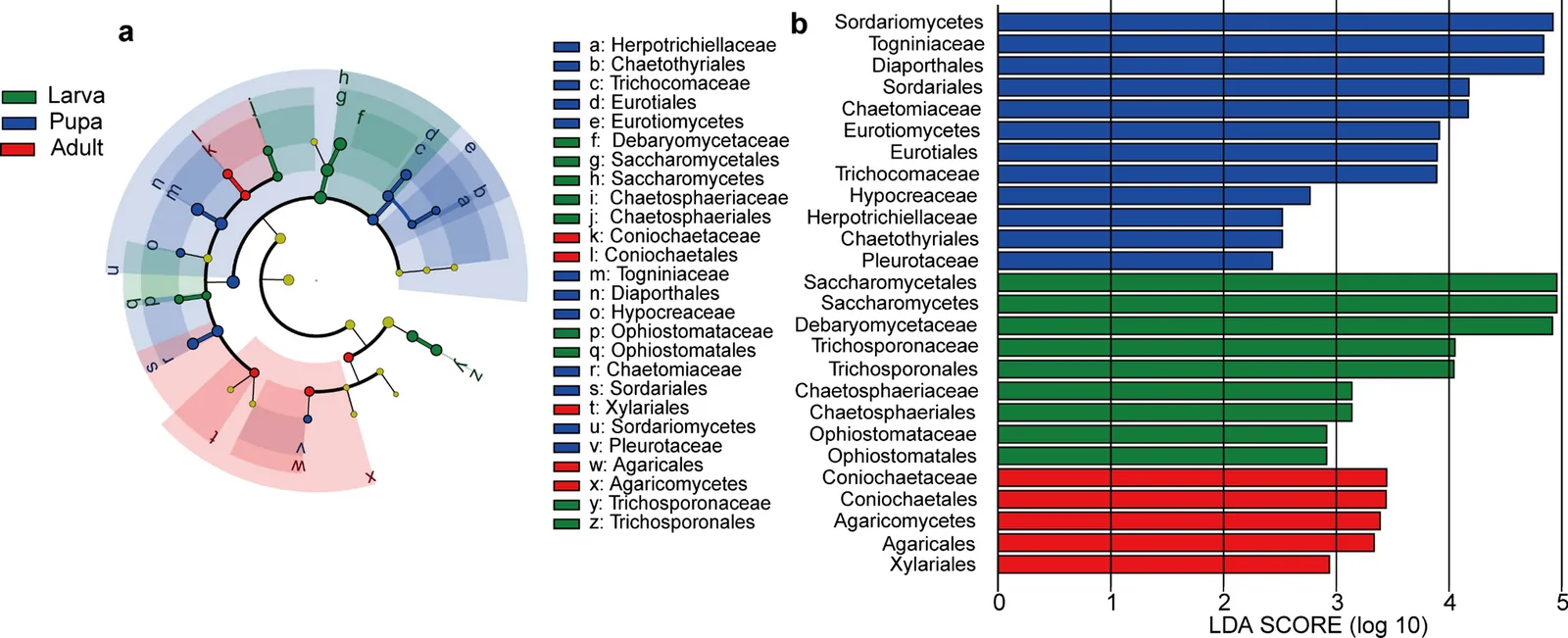
LEfSe analysis of intestinal fungi at different developmental stages. Cladogram showing phylogenetic distribution of microbial communities across different life stages (a). Yellow nodes represent microbial taxa with no significant difference between different life stages; other color nodes represent microbial taxa significantly enriched at those life stages. Identified phylotype biomarkers ranked by effect size (alpha < 0.05) at different stages (b). Phylotype biomarkers were considered significantly abundant when samples from larval, pupal, and adult stages were compared. Larvae include 1st instar, 2nd instar, and 3rd instar larvae. Adults include callow and mature adults

LEfSe analysis was conducted to identify fungal taxa exhibiting differential abundance at different developmental stages in *D. hopei* at the phylum to family levels (Fig. 4a). Analysis was limited to gut microbial taxa with an LDA score exceeding 2.0 among different groups (Fig. 4b). The constructed cladogram illustrated the phylogenetic distribution of dominant microbial taxa from the phylum to family level across different life stages (Fig. 4a). Notably, nine microbial taxa (one class, four orders, and four families) were enriched in the larval stage, nine microbial taxa (two classes, three orders, and four families) were enriched in the pupal stage, and seven microbial taxa (one class, four orders, and two families) were enriched in the adult stage (Fig. 4).

Indicator species also varied across the different development stages, including one species (*Scheffersomyces henanensis*) in the 1st instar stage, one species (*Ophiostoma protea-sedis*) in the 2nd instar stage, seven species (e.g., *Trichosporon veenhuisii*, *Chloridium virescens var. chlamydosporum*) in the 3rd instar stage, seven species (e.g., *Cryptococcus podzolicus*, *Humicola nigrescens*) in the pupal stage, one species (*Trametes versicolor*) in the callow adult stage, and one species (*Penicillium citrinum*) in the mature adult stage (Table S1). SIMPER analysis indicated that *Scheffersomyces* and *Phaeoacremonium* were the primary contributors to the variance in intestinal fungal community composition among the different developmental stages (Table S2).

### Intestinal Saprotrophs, Pathogens, and Endosymbionts

FUNGuild analysis was employed to categorize gut fungi within *D. hopei* as plant saprotrophs, animal pathogens, or endosymbionts. Results indicated an increase in the diversity of animal pathogens during the larval stages, with a subsequent decline from the pupal to adult stages, as well as an initial increase and later decrease in the relative abundance of animal pathogens. The peak in both diversity and relative abundance of animal pathogens was observed in the third instar stage, showing significant differences compared to the other developmental stages. In contrast, the 1st, 2nd, and 3rd instar larvae displayed significantly higher diversity and relative abundance of saprophytic bacteria compared to the other life stages. These findings suggest that gut fungi in larvae may play a more critical role in facilitating host food digestion than in pupae and adults, as evidenced by the increased diversity and relative abundance of endosymbiotic fungi within their gut (Fig. S3).

### Intestinal Fungal Network Analysis

Co-occurrence network analysis was performed to clarify the interactions among intestinal fungal taxa in *D. hopei* during the six life stages. Generally, most links in the intestinal network were related to Ascomycota (Fig. S4). The fungal networks in larvae contained more nodes and edges than other stages, demonstrating a higher density of interactions within gut microbial communities in the larval stages (Table S3). Networks for pupal and adult stages, however, demonstrated higher average connectivity and graph density, along with shorter network diameters and average path lengths, suggesting more complex community structures in the guts of pupae and adults compared to the simpler structures observed in the larval stages.

## Discussion

Various factors, including diet, developmental stage, and host habitat, influence the structure of the gut microbiome [8, 36, 37]. Insects, in response to varying environmental conditions, have evolved symbiotic relationships with distinct microorganisms at different stages of their life cycle [17]. In the present study, we compared the microbial community composition in *D. hopei* across different life stages using Illumina MiSeq high-throughput sequencing. Diet is a well-established driving force affecting changes in the host’s gut microbiota [38]. In this study, the *D. hopei* larvae were fed fermented wood enriched with *P. geesteranus*, whereas adult stages were fed a jelly-based diet. This variation in diet emerged as a significant determinant of gut microbiota diversity, corroborating the findings of earlier research [7]. Prior research has highlighted the complex interplay between dietary components and gut microbes in insects, with dietary shifts leading to rapid changes in gut microbial composition [39]. Moreover, investigations across diverse insect species have revealed that host-specific factors, such as digestive physiology and immune responses, can exert selective pressure on gut microbial communities, independent of dietary influences [40]. Our results demonstrated no significant differences in the gut fungal community between the third instar larvae and earlier larval stages, nor between the callow adult and mature adult stages. These findings may be attributed to two possible factors: first, the consistency in diet and living environment; second, the lesser quantities of beetle jelly food ingested by callow adults compared to mature adults, resulting in insufficient supplementation of both endogenous and exogenous microorganisms. Furthermore, the metamorphosis experienced by holometabolous insects during the pupal stage, which involves significant restructuring of the intestine and other organs and affects gut microbiota attachment [41, 42], likely plays a role in the observed differences in gut fungal community composition across the larval, pupal, and adult stages of *D. hopei*.

*Scheffersomyces*, *Trichosporon*, *Candida*, and *Phaeoacremonium* were identified as the dominant genera in the gut fungal community of *D. hopei* across various developmental stages (Fig. 2). *Scheffersomyces* exhibited a high and consistent relative abundance during the larval stages, yet its presence significantly diminished in the adult stages (Fig. 2). This genus is also found in other insects, such as *Ceruchus* and *Sinodendron*, suggesting its potential involvement in lignin, hemicellulose, and cellulose degradation [20, 43]. Furthermore, *Trichosporon* and *Candida* are known to ferment cellulose and d-xylose, facilitating the absorption of nitrates, xylose, and cellulose, thereby aiding in larval digestion and nutrient uptake [24, 44] (Fig. 2).

In this study, the relative abundance of endophytic fungi was higher in the larval stage than in other developmental stages (Fig. S3). Prior research has indicated that endophytes contribute digestive enzymes and enhance nutritional quality by providing essential amino acids, vitamins, and sterols to their insect host [45]. Additionally, endosymbiotic fungi assist in the degradation of polymeric structural compounds, such as lignocellulose, and in the synthesis of nutrients [46, 47]. Two hypotheses are proposed to elucidate these observations. First, fermented wood, as opposed to beetle jelly, encompasses a more complex composition and a broader spectrum of microorganisms, resulting in larvae ingesting additional microorganisms from fermented wood during feeding. Second, as fermented sawdust is primarily composed of macromolecular substances such as lignocellulose and hemicellulose, compared to simple substances such as agar and brown sugar in beetle jelly, larvae exhibit increased diversity and relative abundance of endosymbiotic fungi in their gut (Fig. S3). Consequently, variations in dominant fungal genera within the gut of *D. hopei* across different developmental stages may result from differences in dietary intake. Such diversity in fungal communities is essential, facilitating access to a wider array of nutrients, thus supporting growth, development, and reproductive success at each developmental stage.

Our results also showed that gut fungal diversity within *D. hopei* first increased and then decreased across developmental stages, consistent with previous research [7, 17]. This pattern may be attributed to the third instar larvae undergoing rapid growth and development, necessitating increased nutrient intake from their diet in preparation for pupation, while the post-eclosion phase in adults requires reduced food consumption. Additionally, microbial community diversity in the fermented sawdust consumed by larvae is higher than that in the jelly consumed by adults, thereby potentially contributing to higher microbial colonization and fungal diversity in the larval gut. Earlier studies have indicated that gut microbial community assembly is controlled by both stochastic and deterministic processes [48, 49], with the gut environment acting as a selective “island” for microbial communities, favoring the formation of unique compositions [35, 50]. In the current study, analysis of gut fungal communities revealed higher NDVs in adults compared to larvae and pupae, indicating a stronger influence of deterministic processes on gut fungal selection [51]. Gut selection is a crucial factor affecting the composition and diversity of microbial communities, favoring certain microbes while excluding others, thereby leading to changes in microbial community composition and diversity. Consequently, the enhanced selective capacity in adults compared to larvae and pupae may significantly impact the gut fungal community structure and reduce diversity in *D. hopei* adults.

Co-occurrence network analysis indicated that the fungal community within the gut of adult *D*. *hopei* displayed increased complexity, with enhanced network stability, elevated connectivity, and reduced path lengths (Table S3), suggesting more efficient metabolic and informational exchange pathways among fungal taxa and better adaptability and resistance to environmental fluctuations [52]. Adults demonstrate superior adaptability and resilience to unfavorable conditions compared to larvae, highlighting a developmental shift towards a more selectively curated and stable gut fungal community. Results showed that the predominant associations within the co-occurrence network were between Ascomycota and Basidiomycota, which were pivotal in structuring the gut fungal community across developmental stages (Fig. S4). These fungal taxa produce various enzymes in insects, such as xylanase and carboxymethyl cellulase, which impact habitat adaptation by facilitating plant polymer digestion [53, 54].

Taken together, our study elucidated the structure of the intestinal fungal community across different life stages of the stag beetle, *D. hopei*, demonstrating significant differences in fungal composition among larvae, pupae, and adults. These findings enhance our understanding of gut microbes in *D. hopei* and their potential interactions with the host. Notably, fungi such as *Scheffersomyces*, *Trichosporon*, *Candida*, and *Phaeoacremonium*, prevalent in the gut of the beetle, play crucial roles in the degradation of cellulose, lignin, and hemicellulose, potentially supporting growth, development, and reproductive success of the host. These insights contribute to a deeper understanding of the potential roles of gut microbes in host development and provide theoretical support for the sustainable utilization and protection of saprophytic insects from a microbial perspective. However, this study has several limitations, including the need for more detailed analysis of fungal content in diet to assess its impact on the composition of the gut fungal community. Future research should investigate gut microbiota variations in wild populations to understand the impact of natural conditions on the microbial community structure and composition.

## Supplementary Information

Below is the link to the electronic supplementary material.

Supplementary file1 (DOCX 3947 KB)

## Data Availability

All raw data were submitted to the Sequence Read Archive (SRA) of NCBI under accession number PRJNA1063643.
