# Supplementary material for: Investigation of Fungal Community Structure in the Gut of the Stag Beetle Dorcus hopei (Coleoptera; Lucanidae): Comparisons Among Developmental Stages
Source: Microb Ecol. 2024 May 14;87(1):70. doi: 10.1007/s00248-024-02379-y (PMC11090938; doi:10.1007/s00248-024-02379-y)
Supplement: Supplementary file 1 — Supplementary file1 (DOCX 3947 KB) [file 248_2024_2379_MOESM1_ESM.docx]

**Supporting Information**

**
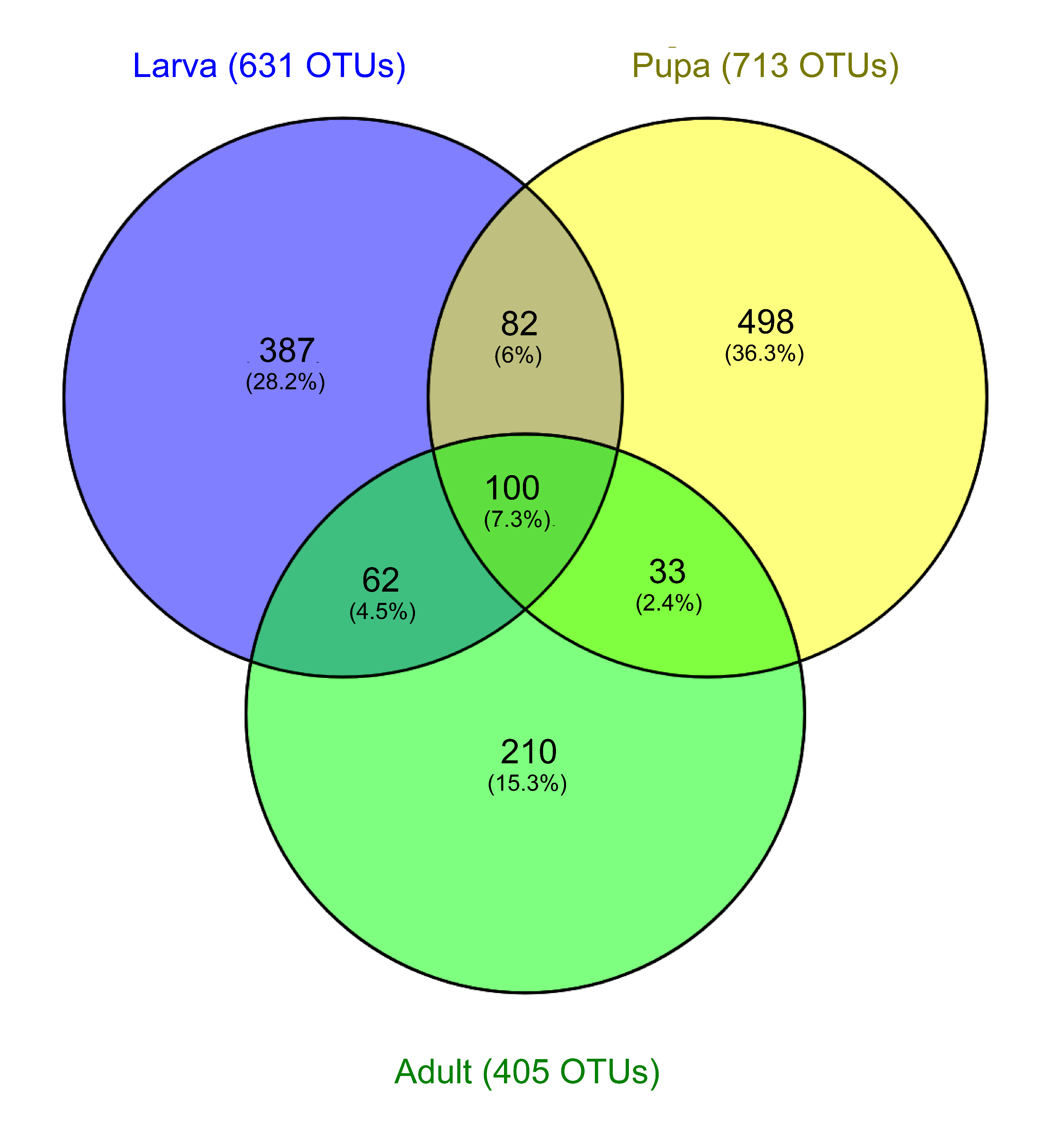
**

**Fig. S1:** Venn diagram showing co-occurrence of OTUs among samples at different stages. Numbers in parentheses indicate total OTUs in each stage group, and numbers inside Venn diagram indicate unique and shared OTUs. OTU, operational taxonomic unit.


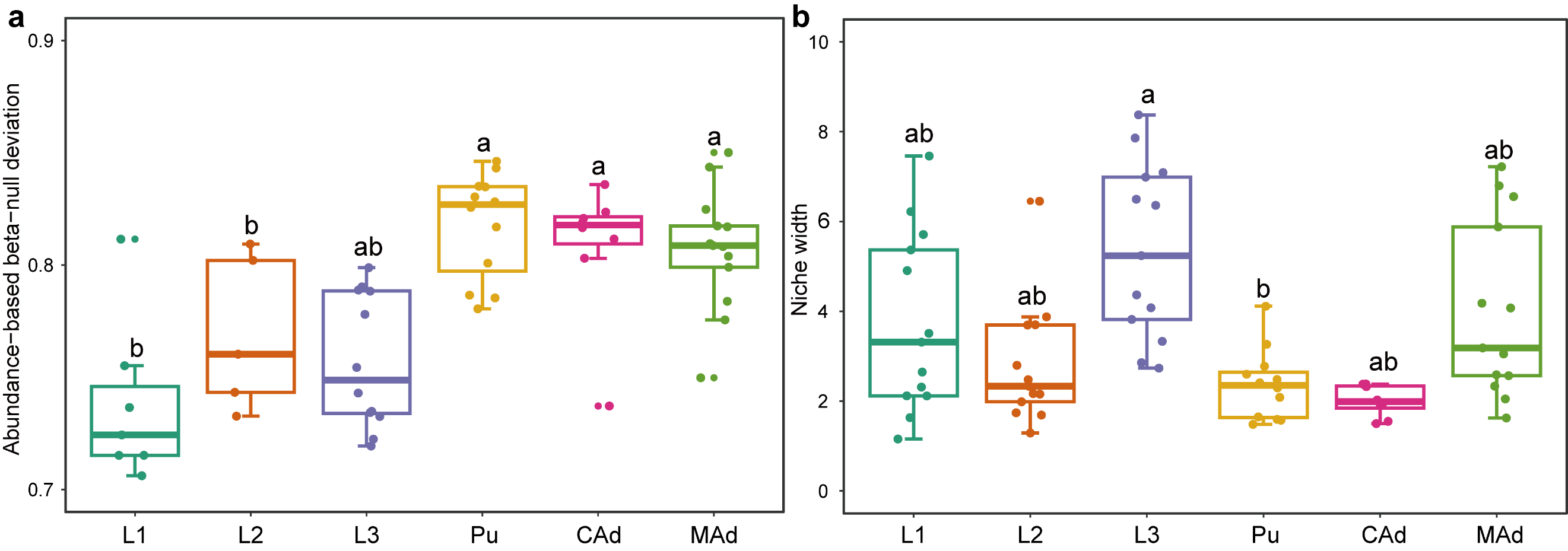


**Fig. S2:** Mechanisms of D. *hopei* fungal community assembly evaluated using abundance-based beta-null deviation and niche width analysis. L1, 1^st^ instar; L2, 2^nd^ instar; L3, 3^rd^ instar; Pu, pupa; CAd, callow adult; MAd, mature adult.


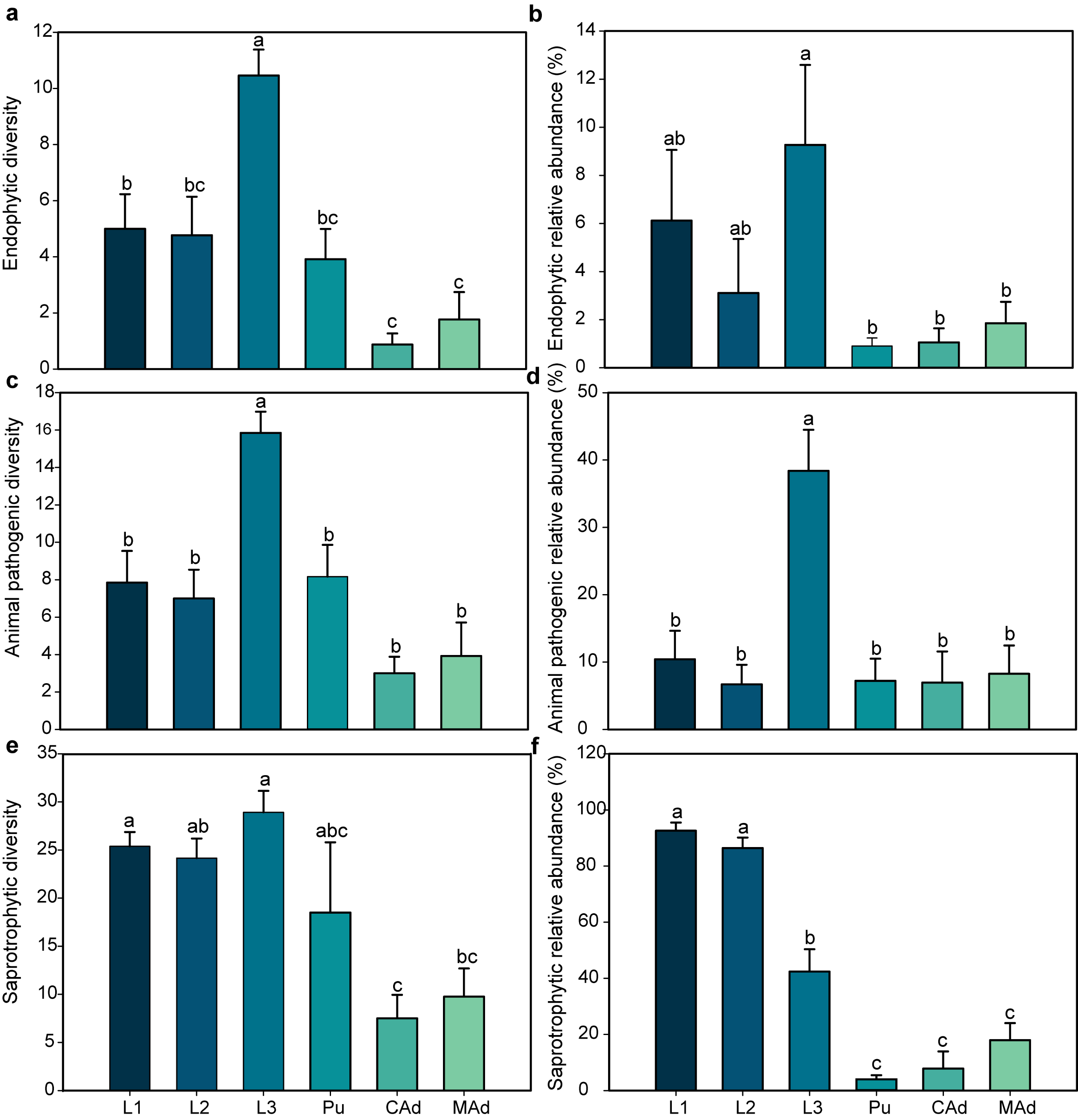


**Fig. S3**: Endophytic diversity (endophytic OTUs) (a); relative abundance of endosymbionts (b); animal pathogenic diversity (pathogenic OTUs) (c); relative abundance of animal pathogens (d); saprotrophic diversity (saprotrophic OTUs) (e); relative abundance of saprotrophy (f). Letters represent significant differences based on Duncan test (*P* < 0.05). L1, 1^st^ instar; L2, 2^nd^ instar; L3, 3^rd^ instar; Pu, pupa; CAd, callow adult; MAd, mature adult.


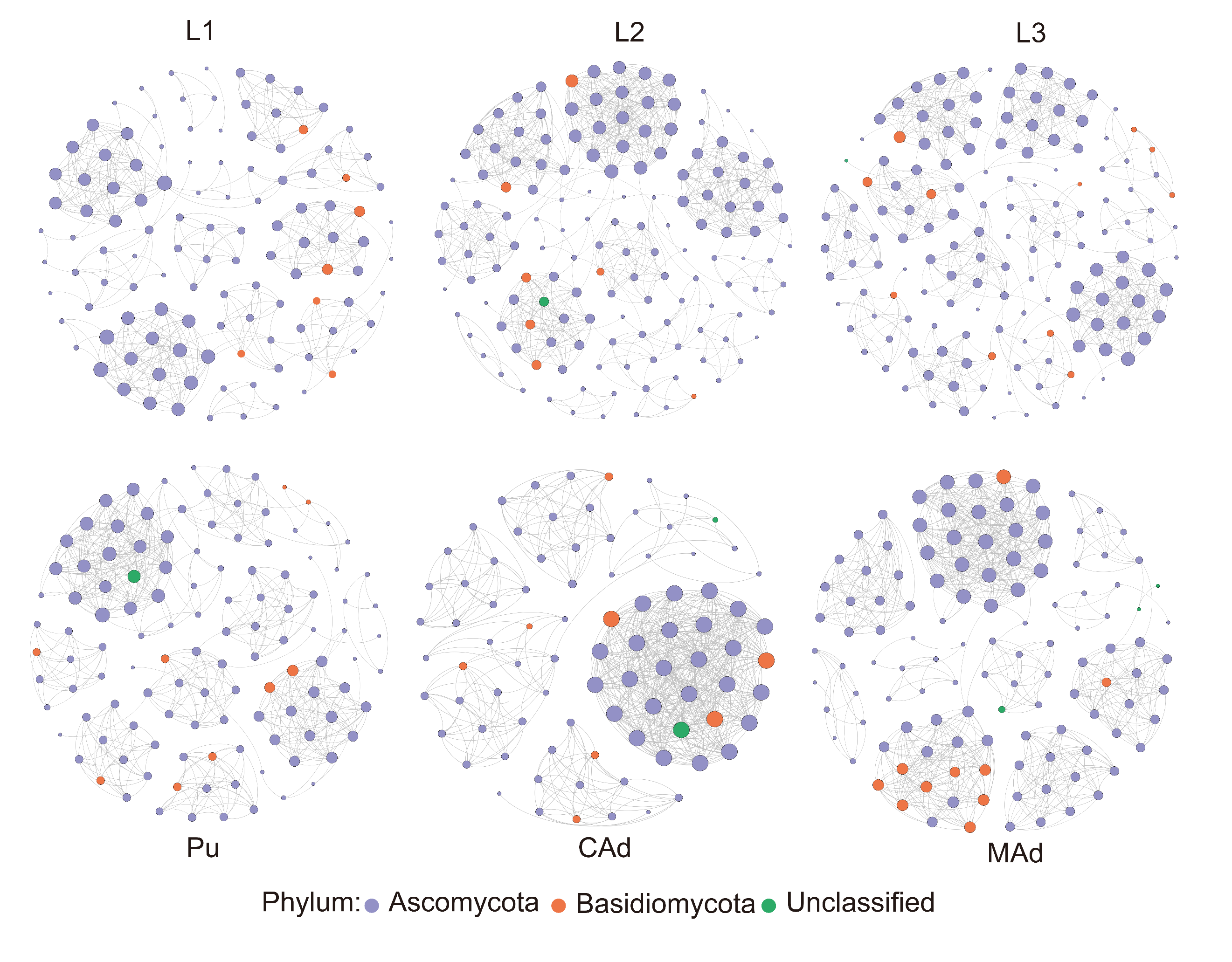


**Fig. S4:** Co-occurrence network structure of gut fungal community in D. hopei, highlighting OTUs with relative abundance greater than 0.01% at the phylum level across six developmental stages. L1, 1^st^ instar; L2, 2^nd^ instar; L3, 3^rd^ instar; Pu, pupa; CAd, callow adult; MAd, mature adult.

| Stage | Taxonomy | Relative abundance (%) | *p* value |
| --- | --- | --- | --- |
| L1 | s_*_Scheffersomyces henanensis* | 44.574 | 0.001 |
| L2 | s__*Ophiostoma protea-sedis* | 0.167 | 0.001 |
| L3 | s__*Scytalidium sp* | 0.005 | 0.033 |
|  | s _*Trichosporon veenhuisii* | 8.491 | 0.001 |
|  | s__*Chloridium virescens* var *chlamydosporum* | 0.059 | 0.002 |
|  | s__*Penicillium charlesii* | 0.007 | 0.011 |
|  | s__*Sugiyamaella novakii* | 0.025 | 0.001 |
|  | s__*Chloridium virescens var chlamydosporum* | 0.017 | 0.012 |
|  | s__*uncultured Dermateaceae* | 0.063 | 0.028 |
| Pu | s__*Cryptococcus podzolicus* | 0.057 | 0.008 |
|  | s__*Humicola nigrescens* | 0.021 | 0.015 |
|  | s__*Mycothermus thermophilus* | 0.005 | 0.03 |
|  | s__*Diatrypaceae sp* | 0.004 | 0.03 |
|  | s__*Lectera longa* | 0.015 | 0.015 |
|  | s__*Fonsecaea pedrosoi* | 0.002 | 0.004 |
|  | s__*Waitea circinata* | 0.003 | 0.044 |
| CAd | s__*Trametes versicolor* | 0.011 | 0.02 |
| MAd | s__*Penicillium citrinum* | 0.213 | 0.029 |

**Table S1:** Indicator species with relative abundances > 0.1% at each developmental stage. L1, 1^st^ instar; L2, 2^nd^ instar; L3, 3^rd^ instar; Pu, pupa; CAd, callow adult; MAd, mature adult.

| **Taxa** | **Contribution (%)** | | | | | | | | | | | | | | |
| --- | --- | --- | --- | --- | --- | --- | --- | --- | --- | --- | --- | --- | --- | --- | --- |
|  | L1 vs L2 | L1 vs L3 | L1 vs Pu | L1 vs CAd | L1 vs MAd | L2 vs L3 | L2 vs Pu | L2 vs CAd | L2 vs MAd | L3 vs Pu | L3 vs CAd | L3vs MAd | Pu vs CAd | Pu vs MAd | CAd vs MAd |
| g__*Scheffersomyces* | 45.40 | 52.60 | 49.70 | 51.00 | 51.00 | 51.60 | 49.50 | 50.80 | 47.50 | 21.90 | 23.00 | 20.20 | 7.80 | 8.00 | 12.10 |
| g__*Candida* | 18.90 | 9.90 | 3.70 | 5.60 | 3.90 | 9.20 | 2.30 | 4.60 | 2.40 | 2.40 | 6.40 | 6.40 | 6.60 | — | 5.60 |
| g__*Trichosporon* | 16.0 | 25.20 | 5.00 | 3.00 | 4.90 | 26.30 | 4.80 | 2.80 | 4.80 | 4.80 | 19.70 | 21.20 | 8.60 | 9.60 | 5.90 |
| g__*Phaeoacremonium* | 13.20 | 10.30 | 32.60 | 36.90 | 29.60 | 10.50 | 33.60 | 37.80 | 30.80 | 40.20 | 40.20 | 35.60 | 55.10 | 47.80 | 50.20 |
| g__*Ophiostoma* | 1.80 | 0.50 | — | — | — | — | — | — | — | — | — | — | — | — | — |
| g__*Aspergillus* | **—** | — | 3.60 | 0.40 | — | — | 3.70 | — | — | — | 4.60 | — | 7.50 | 6.70 | — |
| g__*Mortierella* | **—** | — | — | — | 2.30 | — | — | — | 2.40 | 2.40 | — | 2.90 | — | 4.40 | 4.00 |
| g__*Sporothrix* | **—** | — | — | — | — | 0.70 | — | 0.40 | — | — | — | — | — | — | — |

**Table S2:** SIMPER analysis in fungal community composition at different life stages. L1, 1^st^ instar; L2, 2^nd^ instar; L3, 3^rd^ instar; Pu, pupa; CAd, callow adult; MAd, mature adult.

**Table S3:** Co-occurrence network topological features. L1, 1^st^ instar; L2, 2^nd^ instar; L3, 3^rd^ instar; Pu, pupa; CAd, callow adult; MAd, mature adult.

| Network properties | Group | | | | | |
| --- | --- | --- | --- | --- | --- | --- |
|  | L1 | L2 | L3 | Pu | CAd | MAd |
| Node | 116 | 145 | 147 | 119 | 86 | 120 |
| Edge | 413 | 725 | 597 | 539 | 590 | 715 |
| Modularity | 0.848 | 0.828 | 0.869 | 0.835 | 0.559 | 0.79 |
| Graph density | 0.062 | 0.069 | 0.056 | 0.077 | 0.161 | 0.088 |
| Average degree | 13.433 | 18.683 | 14.862 | 17.189 | 27.237 | 22.602 |
| Network diameter | 8 | 12 | 10 | 5 | 3 | 5 |
| Average weighted degree | 2.313 | 5.628 | 2.716 | 1.645 | 1.020 | 1.874 |
